# Properdin deficiency or anti-properdin treatment ameliorates disease in the C3 gain-of-function mouse model of atypical haemolytic uraemic syndrome

**DOI:** 10.3389/fimmu.2026.1828298

**Published:** 2026-04-28

**Authors:** Grace Mallett, Ola Kamala, Isabel Y. Pappworth, Kate Smith-Jackson, Beth G. Gibson, Muhammad Asif, Harriet Denton, Charlotte Cox, Patrick Walsh, David Kavanagh, Kevin J. Marchbank

**Affiliations:** 1 Complement Therapeutics Research Group, Translational and Clinical Research Institute, Newcastle University, Newcastle-upon-Tyne, United Kingdom; 2 National Renal Complement Therapeutics Centre, The Royal Victoria Infirmary, Newcastle-upon-Tyne, United Kingdom

**Keywords:** aHUS (atypical haemolytic uraemic syndrome), C3 gain-of-function, C3G (C3 Glomerulopathy), complement, kidney or renal failure, mouse model, properdin, therapy

## Abstract

**Introduction:**

We have previously shown that the C3D1115N mouse, engineered around a single point mutation in C3 associated with atypical haemolytic uraemic syndrome (aHUS) in man, fully recapitulates the clinical disease. In this study, we investigated the role of properdin in aHUS.

**Methods:**

C3D1115N mice were crossed onto properdin-deficient mice or C3D1115N mice were treated with anti-properdin monoclonal antibody therapy and survival tracked. Kidney function, serum biomarkers and kidney pathology was carefully assessed at set end points. C3 and Fibrin deposition was assessed using immuno-fluorescence.

**Results:**

We have found that removing properdin ameliorates disease, although two mice had evidence of renal disease over a ~6-month period. Therapeutic treatment with an anti-properdin monoclonal antibody (14E1) after evidence of clinical disease was sufficient to block the progression of disease, suggesting that measures to reduce the stability of the alternative pathway C3 and C5 convertases can help prevent thrombotic microangiopathic anaemia (TMA)/aHUS.

**Discussion:**

Our data indicate that a fine balance exists between disease progression and resolution in our C3 gain-of-function model. The data also suggest that anti-properdin therapy, as noted in another mouse model of TMA, may provide a viable treatment option to maintain remission in complement mediated aHUS.

## Highlights

- Properdin deficiency prevents the development of complement-mediated TMA, suggesting that inhibiting the amplification loop of the alternative pathway of complement and reducing initiation of the terminal pathway is sufficient to prevent clinical disease in the C3 gain-of-function mouse model of aHUS.

## Introduction

The complement system comprises three main activation pathways: the classical pathway, the lectin pathway, and the alternative pathway (AP). These feed into activation of a common terminal pathway that results in the generation of C5a and the formation of the membrane attack complex (MAC). An intact complement system is important for host defence against pathogens, whereas mutations and deletions in key complement regulatory proteins are associated with disease (1). One of the prototypic complement-mediated diseases is atypical haemolytic uraemic syndrome (aHUS) (2), which is characterised by a failure to appropriately control AP activation on the endothelial cells in the kidney, resulting in blood clots (thrombotic microangiopathy [TMA]) and acute kidney failure (3). The treatment of aHUS was revolutionised through the introduction of the monoclonal antibody eculizumab (4, 5) (and subsequently ravalizumab), which targets C5, preventing its cleavage. C5 is at the start of the terminal pathway, with C5b being the first component of the MAC. The MAC plays a key role in killing or controlling certain pathogens (6). Therefore, blocking the terminal pathway completely in patients has risks (i.e., a significant increased risk of meningitis) and is not a panacea (7). Furthermore, eculizumab and ravalizumab are not universally available. This combination of factors has driven a search for alternative anti-complement therapies. Potentially, inhibitors of the AP may be successful in the treatment of complement-mediated aHUS. That said, many of these agents specifically target and bring about complete blockage of the AP (8), which may give rise to a similar risk of pyogenic and meningococcal infections seen with the use of eculizumab (5).

Therapeutic strategies to block the function of properdin may provide a more nuanced approach to the inhibition of the AP. Properdin is a positive regulator of the AP, stabilising both the AP C3 (generating C3bBbP) and C5 convertases (C3bBbC3bP) and, as such, plays a key role in the amplification loop (AL) at the heart of the activation pathway (9, 10), but it is not strictly essential for AP activity (10, 11). That said, properdin is known to play a critical role in the terminal pathway, serving as a dedicated stabiliser of the AP C5 convertase (C3bBbC3bP) and extending its half-life by up to 10-fold through direct binding (9, 12). The AP/AL is responsible for approximately 80% of the terminal pathway activation (13, 14), which drives clinical disease in aHUS. In opposition to the positive effects of properdin on C3 and C5 activation is Factor H (FH). FH is the main fluid phase negative regulator of the AP/AL (15) and competes with properdin when binding to C3bBb and C3bBbC3b (12, 16–18). In combination, it follows that defective FH regulation may enhance the positive regulatory functions of properdin and *vice versa*. Thus, restricting properdin availability in patients with complement-mediated aHUS may be sufficient to maintain either a low or reduced level of complement activation, whilst maintaining great levels of immune defence. This idea was supported by the group of Song who demonstrated that complete blockade of properdin in an aHUS-like mouse model was highly protective (19). These data are the opposite to those noted in the FH and properdin double knockout mouse (a model of C3G) where loss of properdin significantly exacerbated disease (20). Furthermore, data supporting a protective role for properdin in C3G was provided by another model, where low levels of a mutant FH with properdin deficiency resulted in worse outcomes than when properdin was available (21). The reason for properdin deficiency not being protective in models of C3G is likely related to the way properdin interacts with C3 at the cell surface versus in the fluid phase, raising questions about the safety of using anti-properdin drugs in patients with certain rare variants in C3 or FH (6).

We previously developed a mouse model with hyperactive C3, termed the C3 gain-of-function (GOF) mouse (also known as C3^D1115N/D1115N^ or C3^N/N^ for short) (22). The C3^N/N^ mice exhibit renal disease associated with significant AP dysregulation (systemic changes in C3 and C5 turnover as well as significant C3 deposits in the kidney). Animals exhibited spontaneous TMA associated with significant microangiopathic haemolytic anaemia (MAHA), culminating in the death of greater than 50% of animals within the first 10 weeks of life, which could be rescued via C5 and C7 deficiency (22, 23). These data validate the C3^N/N^ mouse model of complement-mediated aHUS for testing anti-complement drugs.

Therefore, the aim of this study was to provide further evidence that depletion of properdin could be a treatment option for complement-mediated aHUS, in the context of a C3 GOF mutation. Our data indicate that properdin deficiency is protective in the C3^N/N^ mouse model of aHUS. Use of anti-properdin monoclonal antibody therapy, once disease was detectable, suggested that depletion of properdin could prevent mice progressing to end-stage renal failure and, thus, provides further confirmation that approaches to reduce properdin expression can reduce the risk or slow the progression of complement-mediated aHUS.

## Methods

### Mice

All mice were housed in a controlled environment (12-h light/dark cycle, 21 ± 2°C) with *ad libitum* access to standard chow diet and water. C3^N/N^ mice were genotyped as previously described (22). Properdin knockout mice [P^−/−^ (24);] were obtained from Prof. Cordula Stover (Leicester, UK) and were re-derived from spermatozoa by Charles River (Lyon, France) before transfer to the animal facilities of Newcastle University. C3^N/+^ were backcrossed for four generations onto the P^−/−^ background. C3^N/+^.P^−/−^ were then intercrossed to produce the C3^N/N^.P^−/−^ and C3^+/+^.P^−/−^ control lines. C3^N/N^.P*^+/^*^−^, C3^N/N^.P^+/+^, and C3^N/+^.P*^+/^*^−^ were also developed during the breeding programme as further controls and experimental animals. Deletion of the properdin gene was monitored using standard ear punch digest PCR including the following primer pairs: WT 5′-GGATTATCACATACTCGTTGACGG-3 with 5′-CTCTTGAGTGGCAGCTACAG-3, and P^−/−^ 5′-CGTGCAATCCATCTTGTTCA-3 with 5′-CAAGGCAGTCTGGAGCATGC-3, 30 cycles (annealing 56°C), generating 1,000-bp (WT) and 500-bp (P^−/−^) cDNA bands. All experiments were given local ethical [Animal Welfare and Ethics Review Board (AWERB), Newcastle University, UK] and UK Home Office approval. Experiments were carried out under the auspices of UK project licences PD86B3678 and PP2560803, with ongoing study plan review.

### Mouse clinical monitoring and terminal blood analysis

Mice were monitored daily (using clinical scoring including weighing and urinalysis—Combistix™, Seimens) from weaning, reducing to weekly from 2 months of age, in the absence of detectable clinical disease. Mice that showed evidence of disease were given soaked diet to mitigate against adverse effects of renal injury. Mice reaching clinical endpoint as guided by the clinical score sheet (reviewed and previously agreed with AWERB) were euthanised and tissues were harvested. Mice that remained clinically well were culled in aged cohorts at 3 and 6 months of age. Where possible, blood was collected using cardiac puncture under 3% isoflurane anaesthesia, followed by cervical dislocation. Blood was collected into pre-coated lithium heparin syringes (in some cases, mice were found in crisis, with insufficient blood volume/haemodynamic pressure to allow blood draw during terminal cardiac puncture). Where available and possible, blood urea nitrogen (BUN) and haemoglobin (Hb) measurements were obtained using an i-STAT1 analyser with a CHEM 8+ cartridge, following the manufacturer’s instructions (Abbott Laboratories LTD). Alternatively, if the iSTAT failed to give a reading, urea was measured on –80°C stored plasma samples using a urea assay kit according to the manufacturer’s instructions (BA0050, Assay Genie, Dublin, Eire).

### Therapeutic properdin inhibition

We tested the ability of the anti-properdin monoclonal antibody 14E1 (25) (gift from Prof. Wen-chao Song and SK Kim, Alexion, New Haven, USA) or the hamster anti-mouse monoclonal H4 (26) (gift from Prof. Dennis Hourcade, Washington University, St Louis, USA) to treat animals after clinical evidence of disease became apparent [2 consecutive days at least 25 erythrocyte per microlitre (ery/µL) haematuria (haem)]. An isotype control antibody was used in randomly selected littermate controls using a freely available coin toss application. To minimise potential confounders, all experimental mice were housed adjacent to each other (either the same cage/or the same row of rack). Mice were given an injection of 1.5 mg of therapy intraperitoneally on day 0 and then 40 mg/kg every 72 h for 8 days. Mice received twice daily health checks during the experiment. Any mouse reaching a predefined clinical score for renal disease or because of general welfare concerns was euthanised during the study (as above), with the remaining culled at the end of the study where blood (as above) and tissue (as described below) were harvested.

### Flow cytometry for reticulocyte and platelet count

To measure reticulocytes, 10 µL of heparinised blood was mixed with 1 mL of phosphate-buffered saline (PBS) control or 1 mL of BD Reticulocyte agent (BD Retic-Count TM, BD Bioscience) for 30 min. To measure platelets, 2 µL of heparinised blood was mixed with 80 µL of PBS, then 10 µL of this solution was diluted in 400 µL of FACS Flow buffer (PBS containing 5% w/v BSA, 1 mM EDTA, and 0.1% w/v Na azide plus 1 µL of purified Rat Anti-Mouse CD41 clone MWReg30 BD Bioscience) for 1 h on ice. Platelet count was established using the method previously described (22). Samples were immediately analysed on a FACSymphony (BD Biosciences, Oxford, UK). Because of COVID-19 facility restrictions, we were unable to collect platelet and reticulocyte counts for the 6-month C3^N/N^.P^−/−^ cohort.

### Analysis of complement protein levels by ELISA

Where possible, EDTA-plasma was collected at the point of mouse euthanasia and immediately placed on ice. After a 5-min spin at 10,000 *g* in a pre-cooled centrifuge, the samples were all stored in multiple aliquots at −80°C, to allow bulk analysis, i.e., samples went through one freeze–thaw cycle before analysis. Briefly, 96-well Maxisorp plates were coated with 1 µg/mL of 11H9 (Total C3; Hycult, Uden, The Netherlands) or mAb 3/26 (Activated C3, specifically C3b, iC3b, C3c, and Hycult) or BB5.1 (Total C5; Hycult) in 0.1 M carbonate buffer, pH 9.6, overnight at 4°C. After washing, plates were blocked with a 3% BSA/PBS solution and then a 1/1,000 dilution of mouse plasma or specific controls, in triplicate, was applied in blocking buffer. After further washing, a 1/25,000 dilution of goat anti-mouse C3-HRPO (C3 assays; MP biologicals) or goat anti-human C5 (Total C5; Comptech, Tyler, Texas) was applied for 1 h at room temperature, as appropriate. For total C5, after washing, a donkey anti-goat-HRPO conjugated antibody (705-036-147-JIR, via Stratech Scientific, UK) was applied at 1:200 dilution. A ready-made TMB substrate (Leinco, UK) was used to develop the assay, which was stopped by the addition of 10% H_2_SO_4_ at 5 min (C3 assays) and 10 min (Total C5). Mouse C5a was detected using a DuoSet ELISA kit following the manufacturer’s instructions (Biotechne, R & D Systems, DY2150). Plates were then analysed on an LT-4500 plate reader (Lab Tech, UK) at OD_450_. In all cases, a standard curve was developed using purified proteins [mouse C3b (Comptech, Tyler, Texas); mouse C5 was purified from mouse plasma using an in-house BB5.1 affinity column and C5a was provided with the kit] and blank-subtracted OD values were extrapolated. Initial EDTA volume and subsequent dilution factors were then accounted for to provide an estimation of protein concentration in the collected blood.

### Histology analysis

Kidneys were harvested, fixed in 10% formalin, and then processed and embedded in paraffin. Sections (4 µM) were then cut and stained for with Periodic Acid–Schiff and Martius Scarlet Blue (Atom Scientific, UK) then imaged on an Olympus X microscope. To quantify glomerular injury, a semi-quantitative scoring system was developed on a scale of 0 to 5. A minimum of 12 glomeruli were randomly selected, scored individually, and then averaged per animal. The scoring criteria integrated features of both C3 Glomerulopathy (C3G/MPGN) and TMA as follows: 0 = Normal: Open capillary lumens; thin basement membranes; smooth mesangial matrix; 1 = Mild: Trace mesangial expansion and/or minor focal hypercellularity (of tuft); 2 = Moderate: Distinct mesangial thickening and early lobulation/segmentation (of tuft); 3 = Severe: Advanced lobular remodelling (“cloverleaf” appearance) or significant capillary luminal narrowing; 4 = Global: Total architectural distortion; global matrix replacement (MPGN) or total luminal obliteration (TMA). Finally, an addition score of 1 was added if fibrin-rich clots were detected in the MSB stain to account for acute TMA.

### Immunofluorescence

Methods for C3 staining are essentially as previously described (22), with minor modification. Staining for fibrinogen was as follows: kidneys embedded in OCT were frozen on dry ice. Samples are fixed with acetone and blocked with normal goat serum. Slides were incubated with rabbit fibrinogen antibody (Abcam 1:400) followed by Alexa Fluor 594 goat anti-rabbit (Invitrogen; 1:200). Slides were then stained with Sudan black in 70% ethanol for 20 min at room temperature. Slides were then repeatedly washed in PBS and then imaged after being mounted in DAPI mounting medium. C3 images were taken at ×20 on Leica DM2000 LED using a Leica DFC 7000 T camera. Fibrin images were acquired using a Zeiss Axioscan 7 and processed using OMERO +. Densitometry analysis of glomerular complement or fibrin deposition was performed using ImageJ. This is presented as corrected total glomerular fluorescence (CTGF) = Integrated Density – (area of manually selected glomerulus × mean fluorescence of six background readings), and between 40 and 60 glomeruli were scored for each mouse.

### Statistical analysis

All statistical analyses were undertaken using GraphPad Prism v10. Mantel–Cox was used for survival analysis. For statistical test between two or more groups, a test of normality (Kolmogorov–Smirnov) was undertaken, and when met, an unpaired Student’s *t*-test was performed. If unmet, then a Mann–Whitney test was used. Welch’s correction was applied if two groups were not assumed to have the same standard deviation (SD). Two-way analysis of variance (ANOVA) with Tukey’s multiple comparison test was used to establish significance between groups and across time. A *P* value <0.05 was taken as statistically significant. *P* values are identified as follows: ns = non-significant, *p < 0.05, **p < 0.01, ***p < 0.005, ****p < 0.0001. Data are shown as mean ± standard error of the mean (SEM).

## Results

### Renal disease-free survival is considerably improved in the absence of properdin

**Figure 1:**
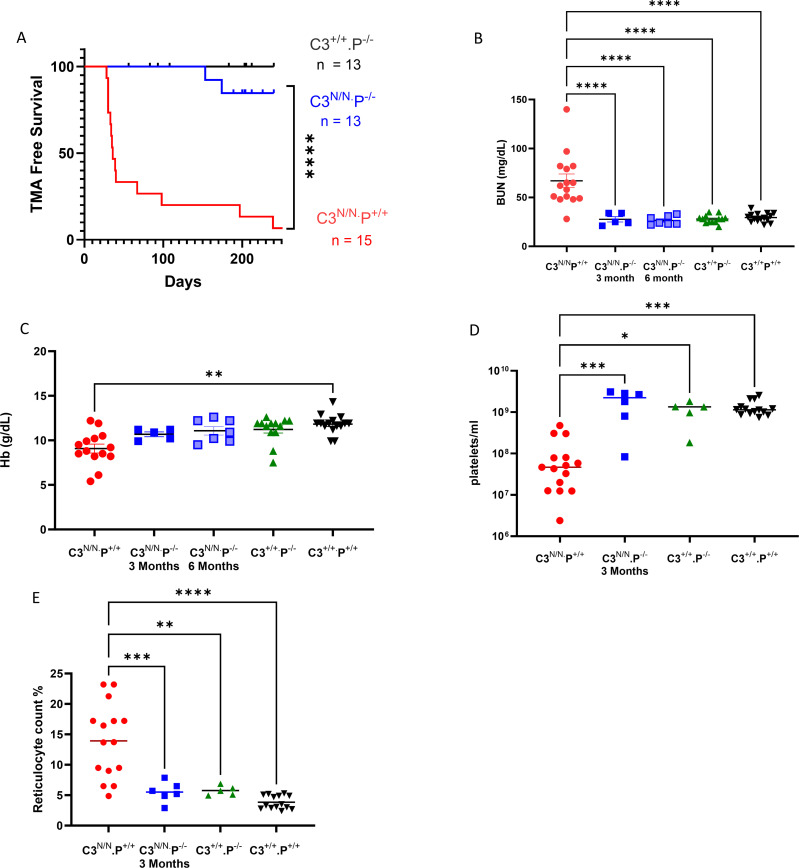
Improved survival from renal TMA in the C3^N/N^.P^−/−^ mice. **(A)** C3^N/N^.P^−/−^ animals were monitored for renal disease (proteinuria/haematuria) from post-partum d15 until they reached 3 (*n* = 6) and at least 6 (*n* = 7) months of age. One animal succumbed to disease with suspected TMA. However, 14 out of 15 C3^N/N^ animals developed TMA during the study. The Mantel–Cox test was used to establish significance. ****p < 0.0001. **(B–E)** Where available, mouse blood was analysed via iSTAT and flow cytometry (see Methods). Genotype is indicated on the *x* axis; *N* varied across the cohorts between 5 and 12 mice according to availability. Values from individual mice are represented by dots, with mean ± SEM illustrated. Two-way ANOVA with Tukey’s multiple comparison test was used to establish significance between groups of mice and across time. Only significant results between the multiple comparisons are shown, with *p < 0.05, **p < 0.01, ***p < 0.005, ****p < 0.0001.

**Figure 2:**
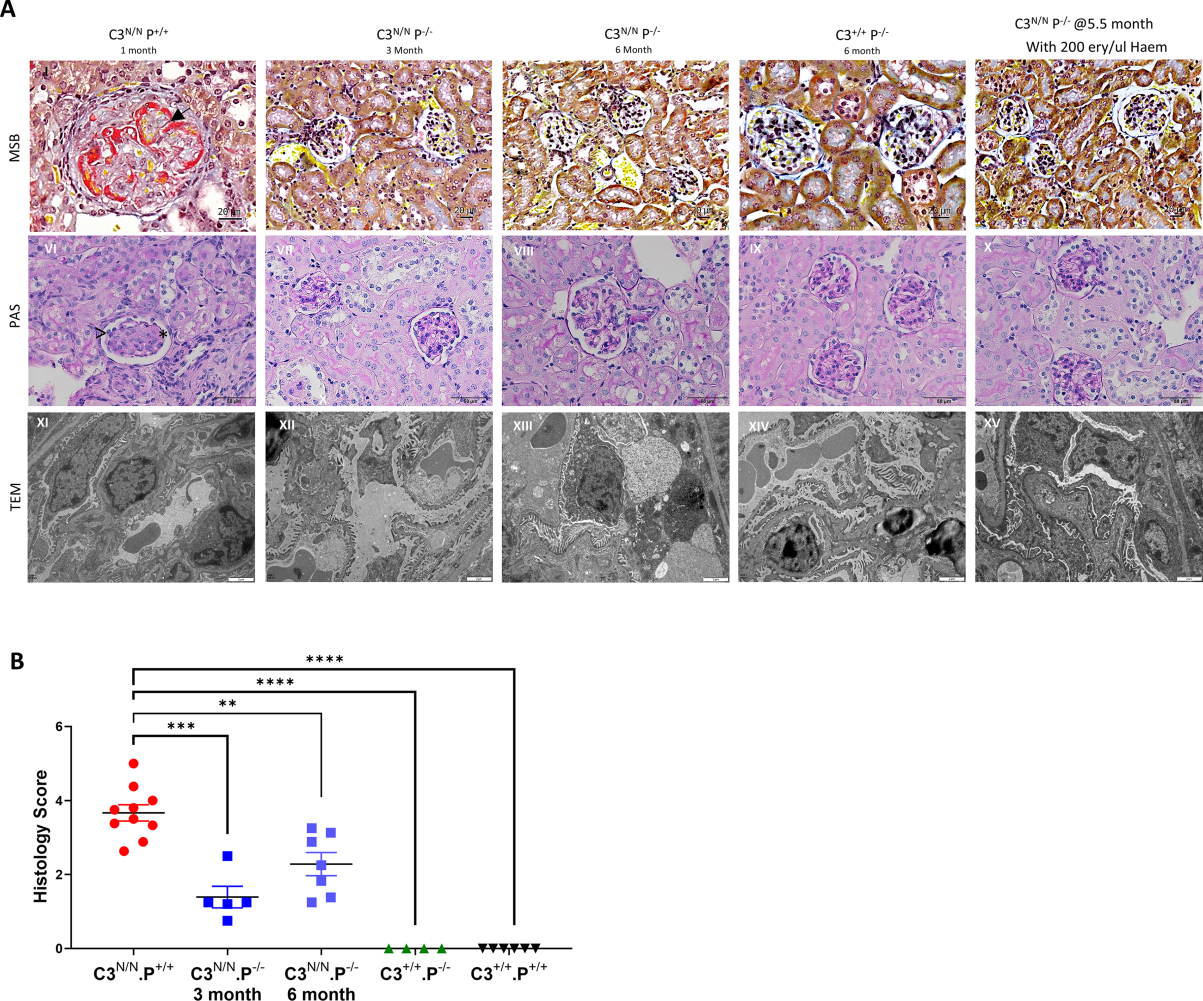
Significant reduction in renal thrombotic microangiopathy in properdin-deficient C3^N/N^ mice. **(A)** Representative images from a C3^N/N^.P^+/+^ (~2 months old), C3^N/N^.P^−/−^ at 3 and 6 months of age, properdin knockout (C3^+/+^.P^−/−^), and the C3^N/N^.P^−/−^ mouse that presented with 200 ery/μL haematuria on day 154. (I–V) Martius scarlet blue-stained sections, (I) arrows showing red fibrin staining within the capillary loops—evidence of microthrombi and thrombotic microangiopathy, congested red cells (in yellow) with irregular and thickened capillary walls, and overall glomerular swelling—none of these features are evident in the other experimental animals (II–V). (VI–X) Periodic-acid–Schiff-stained, (VI) arrows showing evidence of double contouring, mesangiolysis^ and endotheliosis*. (XI–XV) Transmission electron microscopy, (XI) shows evidence of double contouring and podocytes foot effacement, (XII–XIV) no definitive changes are noted but (XV) there is evidence of podocyte flattening, fusion and the GBM appears thickened and irregular in several areas with some evidence of darker, “dense” areas between the endothelium and the GBM, reminiscent of C3G. MSB and PAS, representative images from 10 fields of view across at least six animals per group (i.e., 60 images per genotype). TEM, representative of two animals per group and six fields of view. **(B)** A semi-quantitative histological score was applied to each mouse (MSB + PAS), using a combined scoring approach that accounted for features of both TMA and C3G (see Methods). Each data point represents the average score of one mouse and genotypes are indicated along the *x*-axis. Only significant results between the multiple comparisons are illustrated, with **p < 0.01, ***p < 0.005, ****p < 0.0001.

Our initial analysis revealed that circulating properdin levels were considerably lower in C3^N/N^ mice than in wild-type B6 controls (**Supplementary Figure 1**). These data suggested that properdin consumption was significant in the C3^N/N^ model of aHUS, and therefore, we sought to investigate the effect of removing properdin in this model. Mice that were C3^N/N^ with a properdin deficiency (C3^N/N^.P^−/−^) show a significant survival advantage when compared to the C3^N/N^ controls (**Figure 1A**) whilst partial knockout of properdin (*P^+/^*^−^) had no impact on mouse survival (**Supplementary Figure 2**). One of 13 animals in the C3^N/N^.P^−/−^ cohort was culled in the study after presenting with 200 ery/μL haematuria during routine monitoring at 154 days. The animal did have low platelets (2×10^8^/mL), but BUN was later established to be in the normal range. Careful histological analysis did not establish evidence of TMA, but some evidence of mesangial expansion and increased cellularity is noted compared to the 6-month-old *P*^−/−^ control (**Figure 2**; Supplementary Data 3). A second animal developed mild proteinuria at 170 days. These data suggest that complete deficiency in properdin is largely protective. Survival data are supported by less renal injury (demonstrated by lower BUN) being noted in the surviving mice (**Figure 1B**; no haematological data were available for the mouse found in crisis). Although haemoglobin levels were not significantly different in the C3^N/N^.P^−/−^ mice compared to positive and negative controls (**Figure 1C**), these were closer to baseline values and the other haematological parameters of MAHA; another hallmark of aHUS was returned to normal limits in the surviving C3^N/N^.P^−/−^ mice (**Figures 1D, E**).

### Properdin deficiency significantly reduces histological evidence of TMA in C3^N/N^ mice

**Figure 3:**
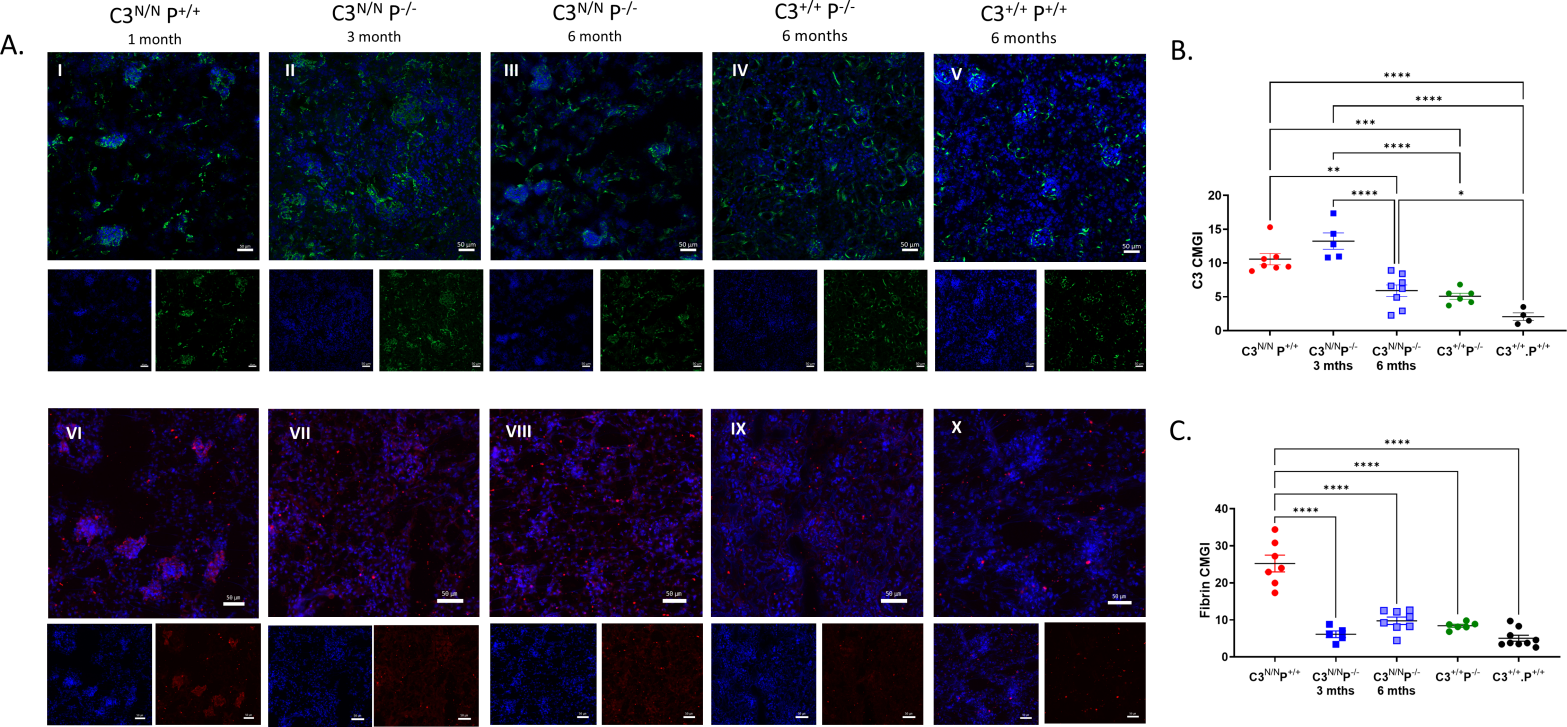
C3 and fibrin staining in properdin-deficient C3^N/N^ mice. Representative immunofluorescence images of C3 (A, I–V) taken on a Leica DM200 at 20× magnification and fibrin (A, VI–X) deposition taken by an Axio7 slide scanner in the various mice (as indicated). The exposure time was kept constant for the individual fluorophores. **(B, C)** Images were saved as LIF files then opened as 8 BIT images in ImageJ and the region of interest (glomerulus) was demarcated, then mean glomerular intensity for this area was calculated within the software. Combined values (average of greater than 40 gloms) from individual mice are represented by dots, with mean ± SEM illustrated. Two-way ANOVA with Tukey’s multiple comparison test was used to establish significance between groups and across time. Only significant results between the multiple comparison are illustrated, with *p < 0.05, **p < 0.01, ***p < 0.005, ****p < 0.0001.

As noted above, only one C3^N/N^.P^−/−^ mouse developed initial evidence of aHUS (haem 200 ery/μL, requiring cull) and low platelets on day 154 of the survival study. However, in all C3^N/N^ properdin-deficient mice, there is no definitive evidence of TMA up to 6 months (**Figure 2**, II, III, VIII, and XIII) compared to a 3-month-old C3^N/N^ properdin-sufficient mouse (**Figure 2**, I and IV), but there is some evidence of mesangial expansion, scarring, tubular stress, capillary wall thickening, and increased cellularity compared to the P^−/−^ control. This visible renal injury does not appear severe enough to produce an elevated BUN level, or the development of a MAHA or even consistent proteinuria (**Figure 1**). As noted above, one C3^N/N^.P^−/−^ ageing mouse demonstrated evidence of renal injury in the routine urine analysis, the mouse presented with proteinuria (~30 mg/dL) on day 170, and endpoint histological analysis is more consistent with glomerulonephritis (**Supplementary Figure 4**) than TMA, although some intraglomerular fibrin clots were detected. In our analysis, three out of seven animals within the 6-month cohort had some histological evidence of C3G (**Figure 2**, VII and VIII). However, deposition of fibrin (as a surrogate for TMA) across the cohort was significantly reduced, to essentially background levels, in C3^N/N^.P^−/−^ mice at both 3 and 6 months (**Figure 3**). Interestingly, C3 glomerular deposition was marginally increased in the kidney of 3-month-old C3^N/N^.P^−/−^ mice, although this increased complement deposition (activation) did not translate to increased proteinuria or significant histological score (**Figures 2B**, **3**) and is reminiscent of the data found with C5 and C7 (22, 23). Whilst glomerular C9 staining was reduced at 3 months in the C3^N/N^.P^−/−^ mice compared to C3^N/N^ controls (1–3 months), it was inconclusive (**Supplementary Figure 5**). Overall, the histological data support a complete absence of TMA but suggest that a C3G/C3GN phenotype can develop over time in a proportion of the C3^N/N^.P^−/−^ mice.

### Properdin deficiency reduces consumption of C3 and C5 in the C3^N/N^ background

**Figure 4:**
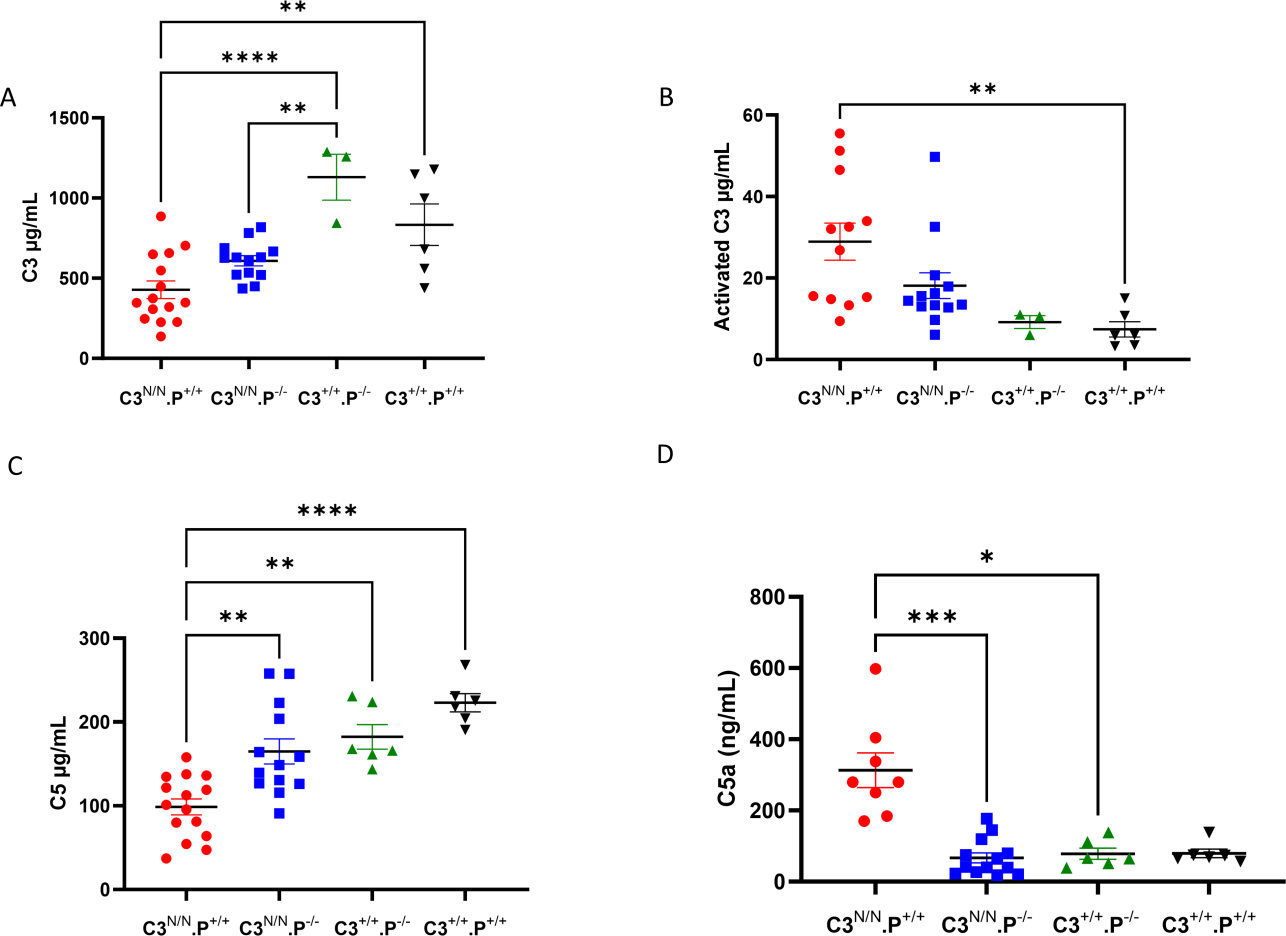
C3 and C5 consumption are restored towards wild-type levels in properdin-deficient C3^N/N^ mice. EDTA-plasma **(where possible)** was collected at euthanasia and stored in multiple aliquots at −80°C to prevent freeze–thaw issues. Samples were analysed using in-house or commercial ELISAs (please refer to Methods). **(A)** Total C3, **(B)** Activated C3, **(C)** Total C5, and **(D)** C5a levels were determined. Each data point represents one mouse and genotypes are indicated along the *x*-axis. *N* varies from 3 to 15 depending on sample availability. Mice included in this analysis were culled at 3 months or, in the case of the C3^N/N^.P^+/+^ mice, taken when they succumbed to disease between 1 and 3 months. Two-way ANOVA with Tukey’s multiple comparison test was used to establish significance between groups and across time. Only significant results between the multiple comparisons are illustrated, with *p < 0.05, **p < 0.01, ***p < 0.005, ****p < 0.0001.

Analysis of total C3 and C5 indicated that properdin deficiency reduced the level of complement consumption in C3^N/N^ mice (**Figure 4**). As noted before (22), significant consumption of both C3 and C5 is a feature of C3^N/N^ mice. In the absence of properdin, this consumption was clearly attenuated. For instance, C3 consumption in C3^N/N^.P^−/−animals^ was not significantly different from wild-type mice, but this effect was most marked with respect to C5 consumption/C5a generation when compared between the C3^N/N^.P^+/+^ and C3^N/N^.P^−/−^ lines. These data may indicate a differential effect of properdin on C5 convertase stability in the C3^N/N^ model compared to C3 convertase stability, offering a potential explanation for the results noted in both **Figures 2**, **3**.

### Therapeutic depletion of properdin attenuates disease progression in C3^N/N^.P^+/+^ mice

**Figure 5:**
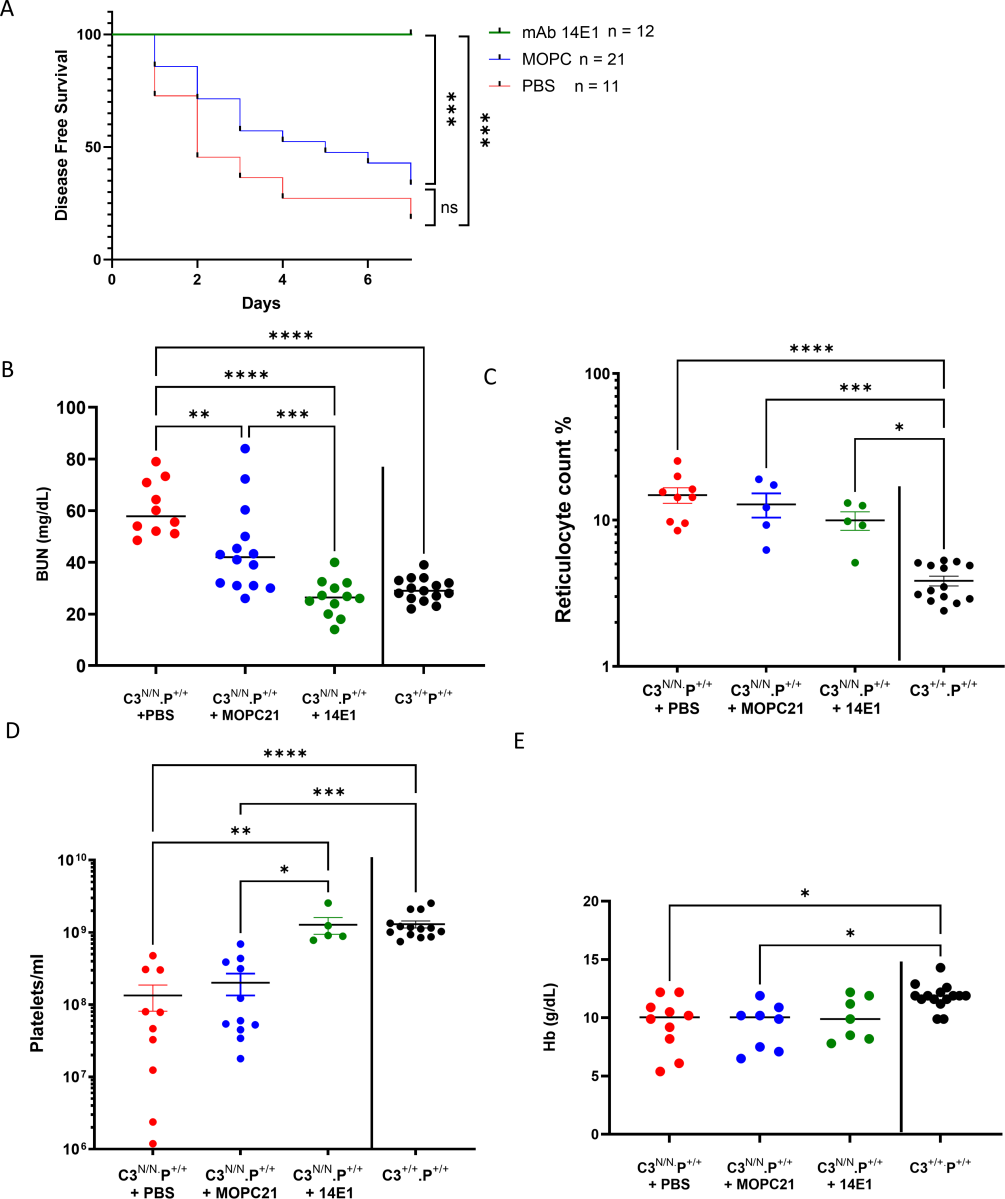
Properdin inhibition in C3^N/N^ mice provides a clear survival benefit and prevents development of clinical disease. C3^N/N^ mice are tracked daily for signs of renal disease from post-partum day 14 by urinalysis; mice with 2 consecutive days of haematuria at 25 ery/μL or greater were randomly assigned to receive either 14E1 or MOPC-31 isotype control antibody at 72-h intervals. Surviving animals were culled on day 8. **(A)** Survival analysis shows C3^N/N^ mice; 2 out of 11 animals receiving only saline succumb to a spontaneous renal TMA within 7 days of clinical disease becoming apparent, and the use of MOPC isotype control antibody is marginally protective with 10 of 21 succumbing to disease, whilst the use of 14E1 was 100% effective in preventing disease progression. **(B–D)** Where available/possible, blood collected at endpoint was subjected to iSTAT analysis for BUN and Hb levels. Platelets were analysed by flow cytometry (see Methods). Two-way ANOVA was used for statistical analysis in GraphPad. Data shown as mean ± SEM. ns, non-significant, *p < 0.05, **p < 0.01, ***p < 0.005, ****p < 0.0001.

**Figure 6:**
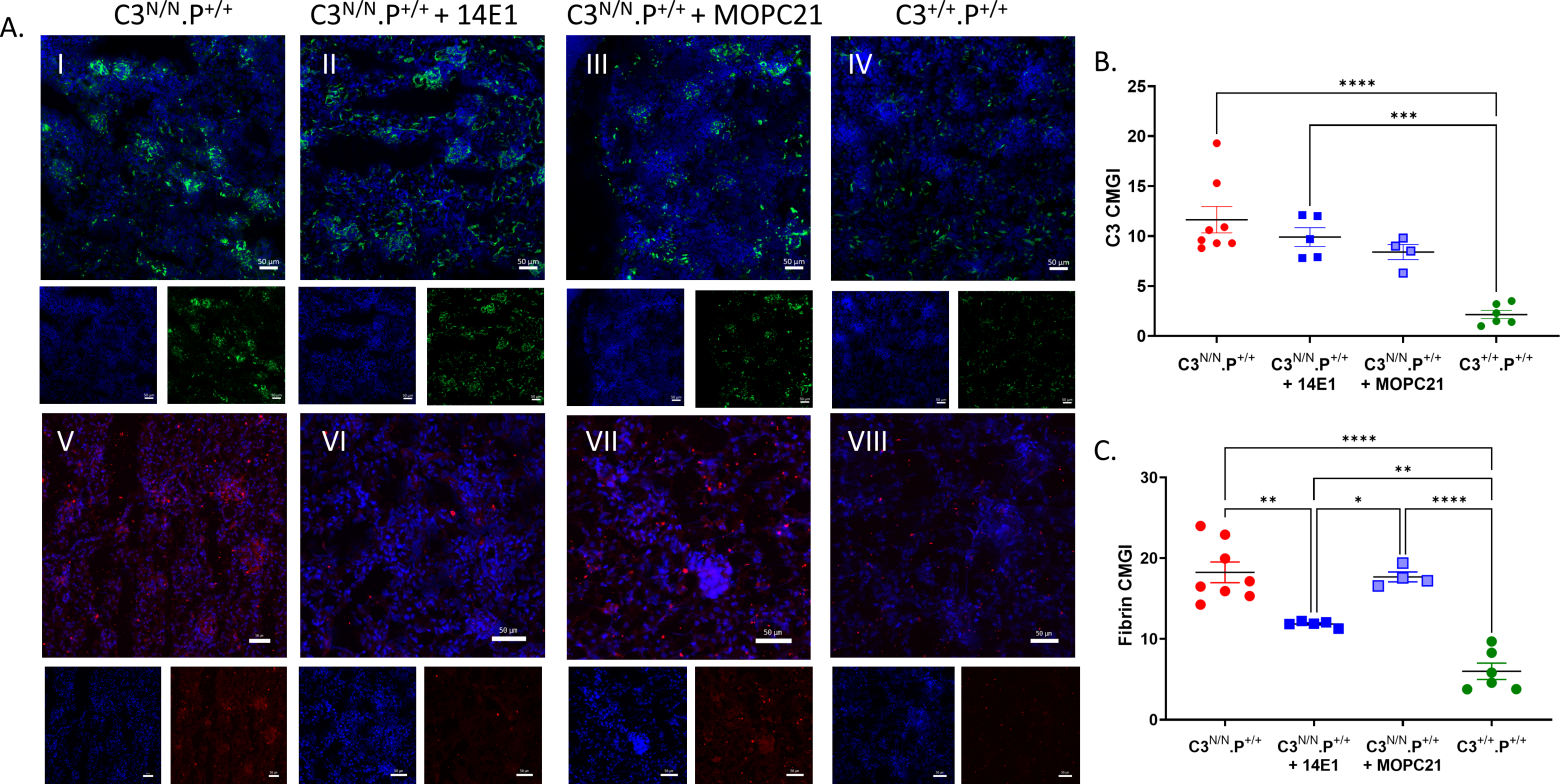
C3 and fibrin staining in C3^N/N^ mice treated with 14E1. Representative immunofluorescence images of C3 (I–IV) and fibrin (V–VIII) deposition in C3^N/N^ mice treated with saline only (PBS), 14E1, or MOPC isotype control antibody (as indicated) taken on a Leica DM200 at 20× magnification or via an Axio7 slid scanner **(A)**. The exposure time was kept constant for the individual fluorophores. Images were saved as LIF files then opened as 8 BIT images in ImageJ and the region of interest (glomerulus) was demarcated; then, mean glomerular intensity for this area was calculated within the software. Combined values (average from greater than 40 glomeruli) from individual mice are represented by dots, with mean ± SEM illustrated **(B, C)**. Two-way ANOVA with Tukey’s multiple comparison test was used to establish significance between groups and across time. Only significant results between the multiple comparison are illustrated, with *p < 0.05, **p < 0.01, ***p < 0.005, ****p < 0.0001.

**Figure 7:**
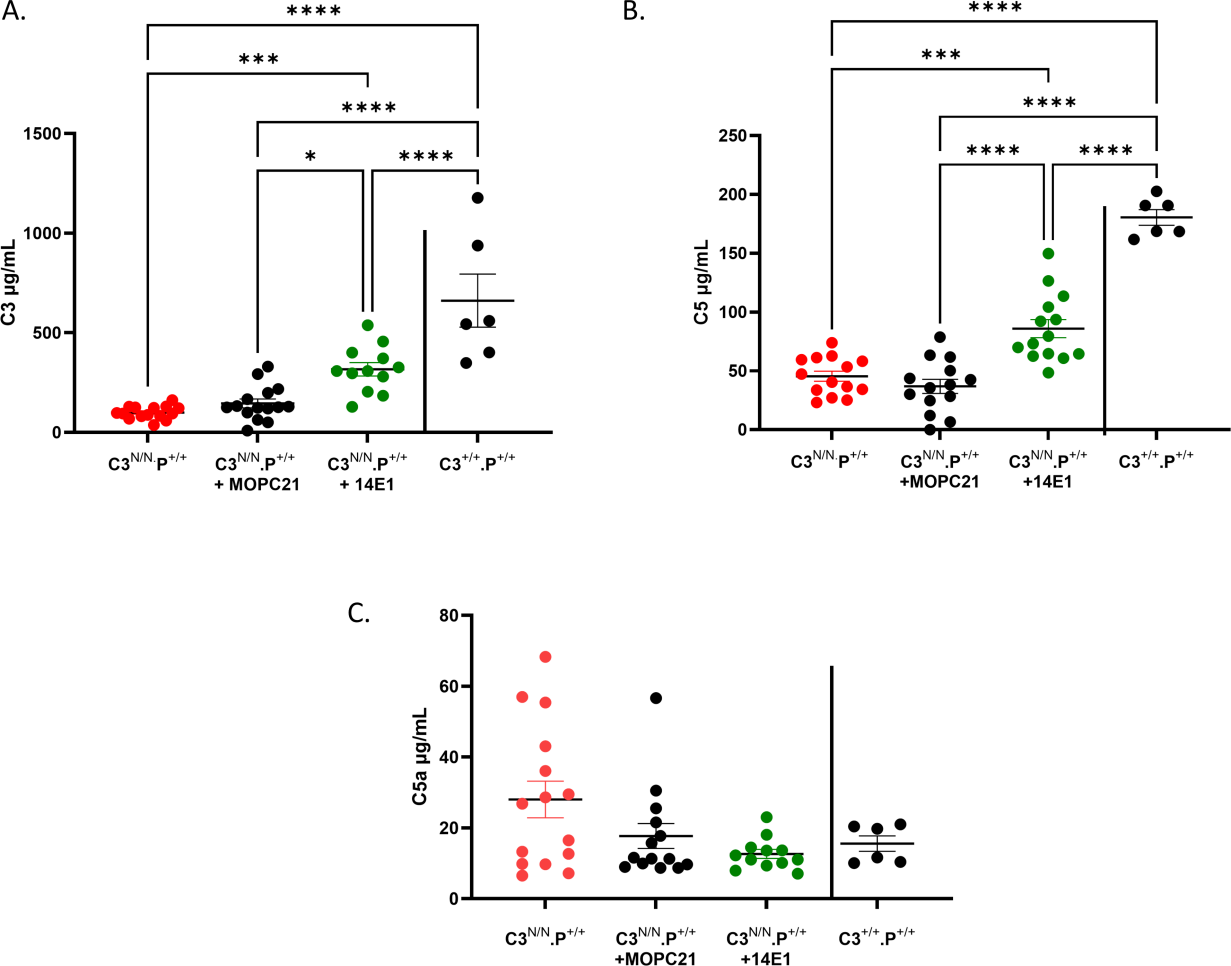
C3 and C5 levels are increased in C3^N/N^ mice treated with 14E1 compared to isotype control. EDTA-plasma (where possible) was collected at euthanasia, processed, snap frozen, and stored in multiple aliquots. Samples were analysed using in-house or commercial ELISAs (please refer to Methods). **(A)** Total C3, **(B)** Total C5, and **(C)** C5a levels were determined. Each data point represents one mouse, and genotypes are indicated along the *x*-axis. N varies from 6 to 15 depending on sample availability. One-way ANOVA with multiple comparison test was used to establish significance between treatment groups. Only significant results between the multiple comparisons are illustrated, with *p < 0.05, **p < 0.01, ***p < 0.005, ****p < 0.0001.

Successful depletion of properdin in mice can be achieved using monoclonal antibodies that target properdin (19, 26). Therefore, we assessed the ability of the hamster monoclonal antibody H4 (26) and the mouse monoclonal antibody 14E1 (25) to protect mice from fatal aHUS after clinical disease was detected, i.e., two consecutive days of haematuria (25 ery/µL, i.e., day –2 and day −1). As mice normally proceed from this stage to humane endpoint (i.e., haematuria at 200 ery/μL) in a few days (22, 23), we opted to use a 1.5-mg dose of monoclonal antibody injected intraperitoneally on day 0 followed by a maintenance dose of 40 mg/kg at 72-h intervals for both monoclonal antibodies [fitting with previous studies (19, 26)]. Mice were tracked for 8 days (culled on day 7) after the first injection. H4 was protective, although three animals did succumb to disease (**Supplementary Figure 6**). We established that whilst H4 (and later 14E1) successfully depleted properdin in the mice (**Supplementary Figure 6**), a rapid immune response to the hamster antibodies developed during the study. This resulted in mouse Ig deposition in the glomerulus and complicated the interpretation of these data (**Supplementary Figure 6**). Therefore, further study with H4 was curtailed. On the other hand, use of 14E1 was 100% successful (*N* = 12) in preventing mice from succumbing to disease with all animals reaching study endpoint with minimal additional evidence of renal disease (**Figure 5A**). Injection of isotype control antibody was also found to have a minor protective effect over saline alone with less than 50% (11/21) of animals succumbing to disease, whilst only 2 of the 11 animals (~20%) given saline only survived within the study time frame (**Figure 5A**). Renal function was significantly improved in mice receiving 14E1 compared to both saline only and isotype control groups (**Figure 5B**). 14E1-treated animals had plasma haemoglobin levels that were not significantly different from control or wild types (**Figure 5C**). However, platelet numbers were fully restored back to those noted in wild-type animals (**Figure 5D**), suggesting a successful block of renal TMA. While the percentage of reticulocytes in the red cell pool was less in the 14E1 treatment group, these remained significantly elevated compared to wild-type levels (**Figure 5E**). These data suggested that the ability of a short treatment with anti-properdin therapy to reverse pathogenicity in the model was mixed and that longer intervention periods may be required to stabilise all parameters. While C3 deposits were essentially unchanged (**Figures 6A, B**), IF analysis demonstrates that anti-properdin treatment using the 14E1 antibody significantly reduced fibrin deposition, although this was not back to wild-type levels (**Figure 6C**). Furthermore, and like the gene knockout, use of the anti-properdin therapy provides clear evidence of a restoration of C3/C5 levels (**Figure 7**), although not completely restoring them to wild-type levels. Overall, the data suggested that depletion of properdin in the C3^N/N^.P^+/+mouse^ was able to prevent the rapid progression of the disease.

## Discussion

We set out to confirm that depletion of properdin would prevent aHUS/TMA in a C3 GOF mouse model and that was clearly established. A key question is why does removing properdin work in this context? Our view is that in the C3^N/N^.P^+/+^ mice, the ratio of properdin to FH [due to its weaker binding to C3 D1115N (22)] is likely shifted at the endothelial surface, allowing excessive C3/C5 convertase formation on the endothelium, leading acutely to the aHUS phenotype. However, when properdin is removed or depleted and despite FH binding to the AP C3 and C5 convertases being compromised in the C3^N/N^.P^−/−^ mouse, FH regulation of the C3/C5 convertase is now sufficient to reduce endothelial surface activation of the AP. Therefore, the conditions required to precipitate a renal TMA are not completely met. However, because regulation of the AP C3/C5 convertases in the C3^N/N^.P^−/−^ mice remains compromised, C3 fragments slowly accumulate in the kidney, eventually manifesting as a mild C3G (no significant proteinuria) detected via the histological analysis. Clear reductions in C5 consumption (C5a generation) in C3^N/N^ mice in the absence of properdin in comparison to more subtle effects on C3 consumption/deposition suggest that a stabilised C5 convertase (at least in this model) is of greater importance for the development of TMA. Furthermore, the data presented herein suggest that properdin is essential for the rapid, high-intensity activation of the AP and AL seen in aHUS, but in its absence, a slower, chronic “smouldering” activation of the AP will still continue to lead to renal injury that is more characteristic of C3G. Whether what we have seen in the C3^N/N^ mouse would be seen in patients with other C3 GOF or FH loss-of-function mutations is difficult to predict, but it is a cause for concern for any long-term use of an anti-properdin therapy in the treatment of aHUS. That said, short-term or targeted local use of an anti-properdin therapy during an acute TMA could be effective. Based on the data herein, short-term usage would likely be sufficient to reduce C3/C5 convertase activity, stall the AL, and reduce terminal pathway activation sufficiently to prevent the conditions required for a renal TMA.

This fits with a growing dataset that now confirms that long-term complete blockade of the complement system (either AP or terminal pathway) is not risk-free (27). The use of animal models of complement-mediated aHUS therefore play an essential part of pre-clinical testing of putative agents. The first described model, FH delta CCP16-20, used a genetic deletion of the last five SCRs of FH, a change not seen in man, to demonstrate that the carboxy terminus of FH is essential to prevent aHUS-like disease (28). A more recent model based on a single point mutation in FH (i.e., FH W1206R, FH^R/R^ mice) found in patients with aHUS presents with many of the pathological changes seen with aHUS in man (19, 29). However, FH^R/R^ mice suffer significant systemic thrombosis and large clot formation in many organs, particularly the liver and brain (29). This is not a feature commonly found in aHUS in man, possibly as homozygous FH^R/R^ patients who might develop macrovascular thrombosis would not survive. However, this is difficult to assess, and therefore, findings in the model, as with any animal model, should be taken with a level of caution. Therefore, the certainty of the mechanism behind the disease-blocking role of properdin deficiency in FH^R/R^ mice (19) remained open. For instance, as the role of properdin is complex (30) and intersects with the activation of the coagulation pathway, it was very possible that the level of effect they noted could have been confined to that particular model, owing to clot modulation and not via direct effects on the AP. All that said, the data herein, in the C3^N/N^ mouse model of complement-mediated aHUS, which mirrors patient clinical features seamlessly (22), confirm that properdin is a modifier of complement-mediated aHUS and supports the findings in the FH^R/R^ model. Furthermore, as suggested above, these studies in combination also appear to support the concept that properdin’s role in stabilising the C5 convertase is a key factor in complement-mediated aHUS and the eventual formation of a TMA. Interestingly, properdin mutations in association with aHUS are extremely rare (31). Of course, to be pathologically important, the changes noted would, in some way, lead to a properdin gain of function, to outcompete or further diminish FH/Factor I function and enhance C5 convertase formation or stability. To our knowledge, no analysis of missense mutations found in properdin in association with aHUS has been carried out, but based on these and other data, it would potentially be an interesting avenue of investigation to follow.

The need for alternative anti-complement drugs in certain complement-mediated diseases and disease states remains pressing (7). The targeting of properdin has several obvious/attractive points (6, 32). For instance, properdin is produced by monocyte/macrophage populations and is found in the circulation at relatively low concentrations of 5–15 µg/mL (much less than C5, at 50–75 µg/mL, for instance). Thus, one of the potential advantages that an anti-properdin treatment might offer is the need for lower doses of the antibody, although that remains to be seen based on the current pre-clinical work. Furthermore, local renal levels of properdin may be rapidly modulated in a disease state, and this could undermine this concept further in a clinical setting. In both the FH^R/R^ and the C3^N/N^ models, a relatively high dose was given, but work from others suggest that lower doses are possible to modulate the AP in mice (33). Both H4 and 14E1 appeared to rapidly deplete properdin efficiently *in vivo* from the blood (**Supplementary Figure 6**). This might be linked to the multimeric nature of properdin and the use of bivalent anti-properdin mAbs, where large immune complex lattices could form, which would be cleared quickly via scavenger receptors. In the case of H4, this may also have contributed to the rapid immune response to the hamster Ig. Thus, the generation of immune complexes does potentially raise concerns for the safe therapeutic use of bivalent anti-properdin antibodies in patients. As previously noted (23), the use of isotype control antibodies in C3^N/N^ mice can provide a therapeutic effect; this is possibly a mixture of plasma expansion and mild complement depletion, being possibly akin to the protective effects associated with IVIg (34).

Our findings also share some similarities with data in FH^R/R^ mice with a C6 deficiency (35), providing further support to the idea that the AP C5 convertase, as well as the activation of the terminal pathway, is responsible for setting the conditions for the downstream pathological injury associated with complement-mediated aHUS (11). The ability of FH to work against properdin on the AP C3 and C5 convertases is likely critical to disease control in the kidney. In the C3^N/N^ mouse, FH binding to the C3b is reduced significantly and is considered to be the main mechanistic change that eventually leads to TMA and aHUS in this model (22). Intriguingly, we noted that FH levels were increased in C3^N/N^.P^+/+^ mice (22). Given that as little as 20% of normal FH levels is sufficient to protect renal surfaces (36) and that the lower levels of FH in that study were still sufficient to restrict AP C5 convertase formation and the consumption of C5, it follows that removing competition with properdin for access to the C5 convertase is likely sufficient to tip the balance in favour of regulation, even when FH binding to the C3b as part of the C5 convertase is compromised. The fact that one key FH function with respect to binding to glycosaminoglycans in the kidney (37, 38) is not affected in either the FH^R/R^ [indeed may be marginally enhanced in this model (19)] or C3^N/N^ mice further supports the potential for FH to exert additional control of the C3 and C5 convertases in the absence of properdin in the renal microenvironment. Furthermore, in the context of complement-mediated aHUS, properdin has been reported to enhance complement activation on activated platelets, which may contribute to complement activation in the kidney of patients with aHUS (39). The consumption of properdin in the C3^N/N^ mice (**Supplementary Figure 1**) may be a reflection of this, and the suggested roles for properdin in sustaining the AP may also contribute to the protective effects of properdin depletion, i.e., reducing the feedback loop of additional complement activation in the damage kidney vasculature because of clot formation.

In light of these results, it adds further credence to clinical trials with anti-properdin therapy in complement-mediated aHUS and other diseases driven by complement and TMA. However, as noted in the Introduction, properdin has been found to have protective roles in certain disease conditions including C3G, atherosclerotic plaque progression (40), and heart failure in man (41), and so these studies remain challenging. Furthermore, and as noted above, the evidence that a C3G/C3GN phenotype is developing in some mice may highlight a limitation for long-term use of an anti-properdin therapy with the caveat that mouse models do not always replicate the human condition and that this C3 mutant (D1115N) may sit close to a tipping point, allowing progression to either C3G or aHUS depending on additional background/environmental factors. This may not be the case in man. We, and others, have previously reviewed the potential of using anti-properdin therapy in patients (6, 32).

Overall, our data confirm that properdin plays a key role in complement-mediated aHUS, likely through its function to stabilise the AP C5 convertase and/or prevent access of FH to the AP C3 and C5 convertases. The use of anti-properdin therapy was effective in the short term and therefore may have clear therapeutic utility in an acute or local setting, where the AP and AL are hyperactive.

## Data Availability

The raw data supporting the conclusions of this article will be made available by the authors, without undue reservation.
