# Supplementary material for: Properdin deficiency or anti-properdin treatment ameliorates disease in the C3 gain-of-function mouse model of atypical haemolytic uraemic syndrome
Source: Front Immunol. 2026 Apr 28;17:1828298. doi: 10.3389/fimmu.2026.1828298 (PMC13160788; doi:10.3389/fimmu.2026.1828298)
Supplement: Supplementary file 1 [file DataSheet1.pdf]

Supplementary data – Properdin deficiency or anti-properdin treatment ameliorates disease in the C3 gain-of-function mouse model of atypical haemolytic uraemic syndrome. Mallett et al, 2026.

https://www.frontiersin.org/articles/10.3389/fimmu.2026.1828298/full#supplementary-material

Supplementary Figure 1.

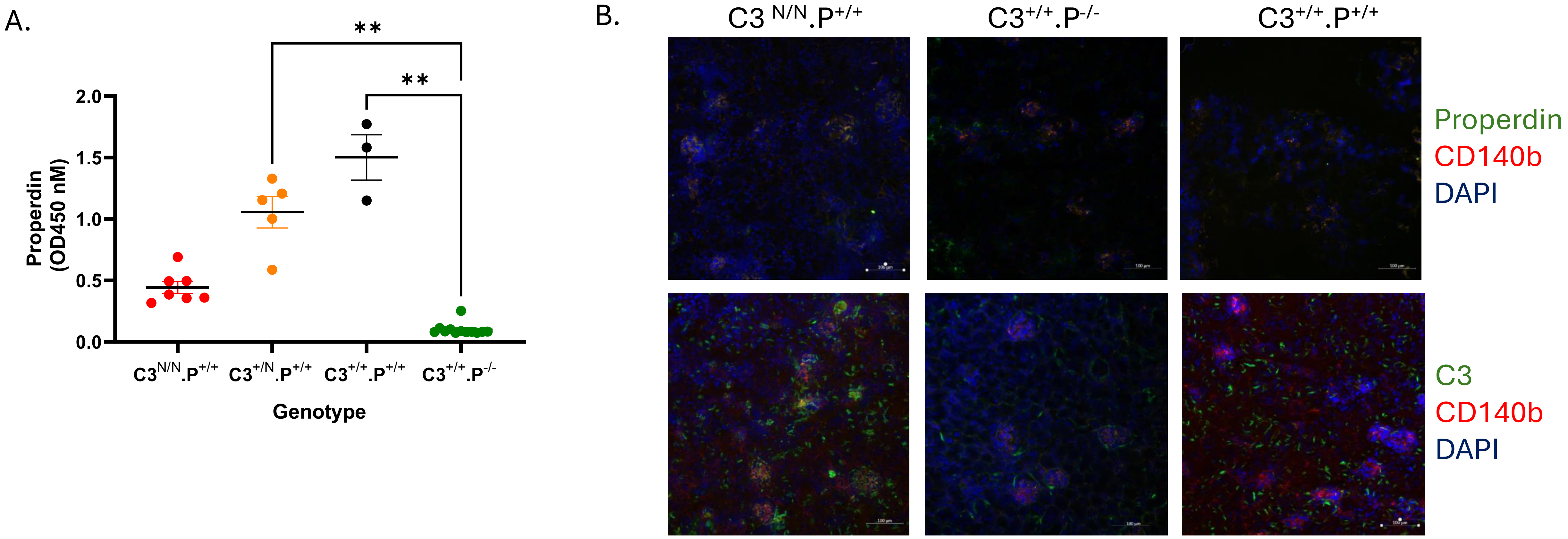

Properdin levels are significantly lower and properdin/C3 associate with the mesangium in $C3^{N/N}$ mice. **(A):** Levels of properdin were analysed from plasma obtained from different phenotypes of our C3 Gain of Function mice using a sandwich ELISA technique (n=7 homozygotes ($C3^{N/N}.P^{+/+}$) , n=5 heterozygous $C3^{+/N}.P^{+/+}$, n=3 $C3^{+/+}.P^{+/+}$ and n = 11 $C3^{+/+}.P^{-/-}$ mice). MaxiSorp ELISA plates (ThermoScientific, NUNC-IMMUNO PLATE) were coated with H4 antibody (Armenian hamster anti mouse properdin) 1:200 in coating buffer (0.2M carbonate/bicarbonate, at pH of 10.6 – 0.95µg H4 per well) overnight at 4˚C. Plates were washed in wash buffer (PBST (Tween 0.1%)) thrice before blocking in blocking buffer (1% BSA in PBST) at 37˚C for 1 hour. Plates were washed thrice and serum/plasma (diluted 1:100 in blocking buffer + 2.5mM EDTA) was added (for plasma tail bleeds, dilution factor was noted to amend final OD reading) and left to incubate at room temperature for 2 hours. After a further three washes, pAb 1209 (Dennis Hourcade, Washington University) was added to each well (diluted 1:25000 in blocking buffer for a final concentration of 6.7ng per well) and left to incubate at 37˚C for 1 hour. Following an additional three washes, goat anti Rabbit HRP conjugated pAb (Jackson Immuno Research 800-367-5296) was added (diluted 1:5000, 10ng per well) incubated at 37˚C for 1 hour. Plates were washed thrice and 50µL TMB (Leinco Technologies, T118) was added to each well and left to develop for 4 min, after which, 50 µL of 10% H_2SO_4 was added to stop the reaction. OD was measured using a plate reader (Labtech.com, LT-4500) at 450nm absorbance. Kurkal-Wallis with Dunn's multiple analysis correction was used to establish significance. ** = P<0.01. **B).** Briefly, kidneys embedded in OCT were frozen on dry ice. After fixation, sections were permeabilised with triton X and blocked with rabbit serum. Slides were incubated with Rat anti-mouse CD140b (or anti-PDGFRB; clone AP85, Invitrogen/eBioscience, UK, 1/200) and hamster anti-Properdin (clone H4, gift from D. Hourcade, 1µg/ml) or anti-C3-FITC (MP Biologicals, 1/200). This was followed by incubation with a goat anti- Armenian Hamster or -Rat IgG (H+L) Cross-Adsorbed Secondary Antibody, Alexa Fluor™ 555 (A78964 or A21434 Invitrogen, UK) as appropriate. Slides underwent repetitive washing in PBS and then imaged after being mounted in DAPI mounting medium. Images were taken at x20 on Leica DM2000 LED using a Leica DFC 7000 T camera.

Supplementary Figure 2.

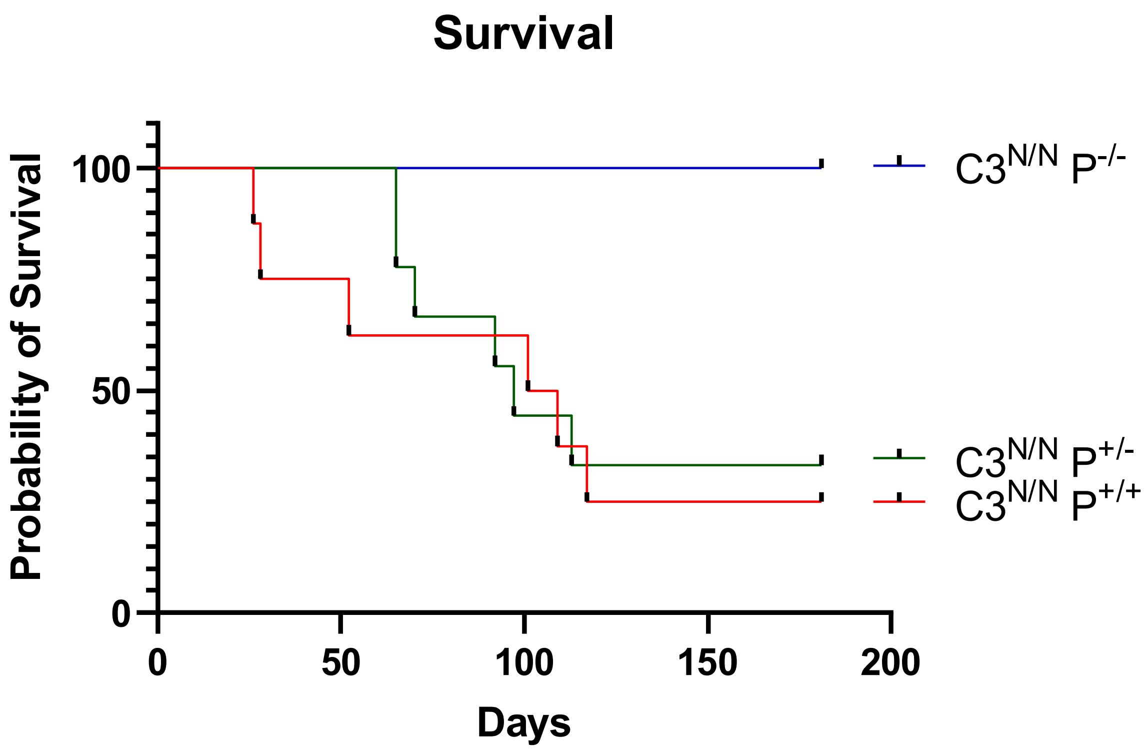

Absolute survival of $C3^{N/N}.P^{-/-}$ (n = 13), $C3^{N/N}.P^{+/-}$ (n = 10) and $C3^{N/N}.P^{+/+}$ (n = 6) mice from post-partum d15 to a maximum of 27 weeks before cull. Heterozygous properdin deficiency is not protective.

Supplementary Figure 3

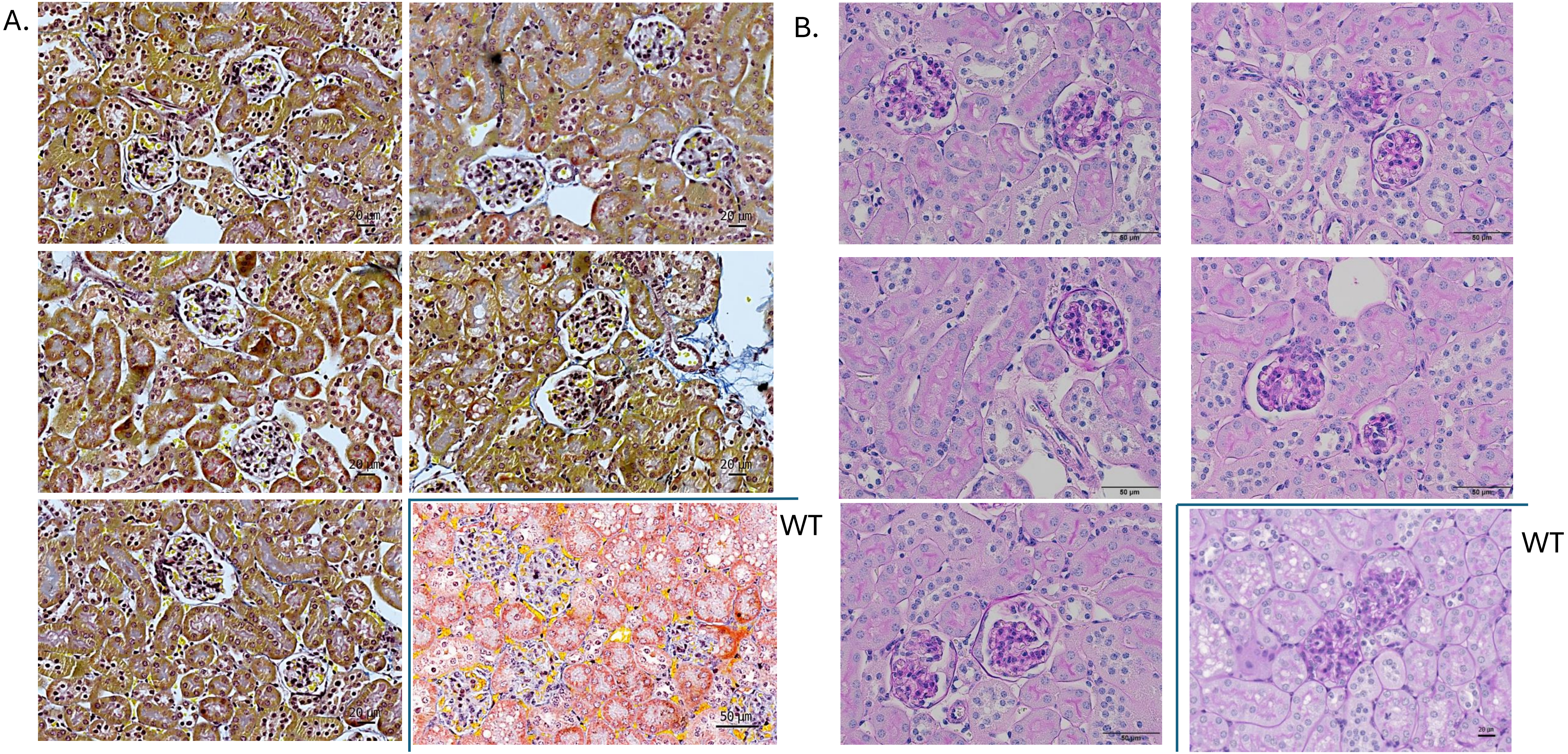

Following the methods in the main article additional representative images from Mouse 9191 – $C3^{N/N}.P^{-/-}$ culled at 154 days following 200ery/µl Haematuria. MSB (A) and PAS (B) staining reveals a mild mesangial expansion and possibly indicates early-stage C3 glomerulopathy (C3G). There is moderate hypercellularity compared to baseline wild type. Capillary walls appear thicker than the WT but nothing to explain the 200 ery/µl haematuria that resulted in the mouse being euthanised. It is possible the haematuria was due to a vascular bleed in the urethra during handling or a UTI, or similar.

Supplementary figure 4

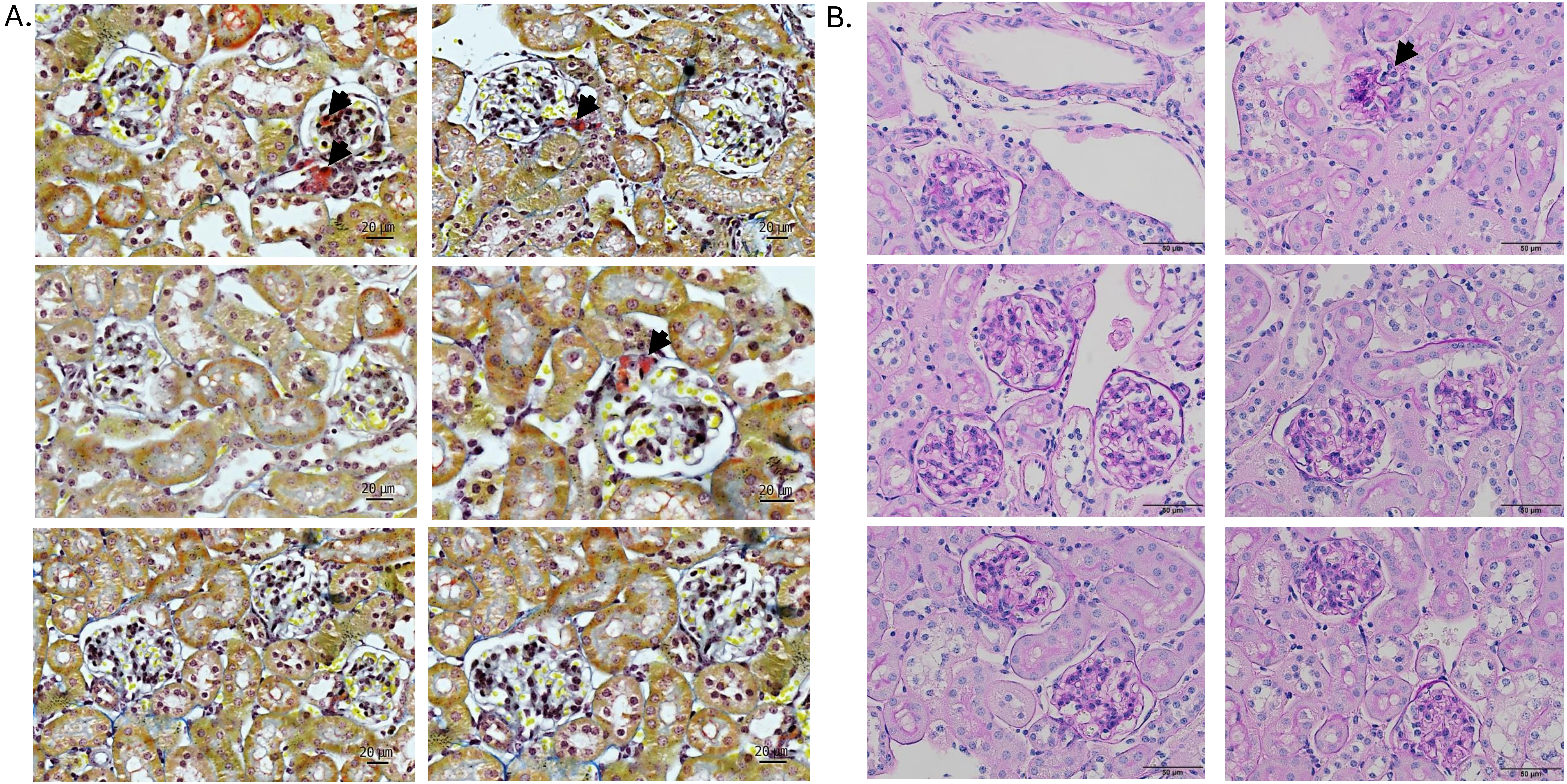

Following the methods in the main article, representative histology from Mouse 5679 – a $C3^{N/N}.P^{-/-}$ mouse that developed mild proteinuria at 170 days. MSB (A) and PAS (B) show evidence of chronic inflammatory responses and a glomerulonephritic phenotype, increased connective tissue, collagen, increased cell infiltrate, interstitial inflammation. Arrows indicated potential intraglomerular fibrin clots in (A), potential congestion and crowding of the glomerulus in (B).

Supplementary Figure 5.

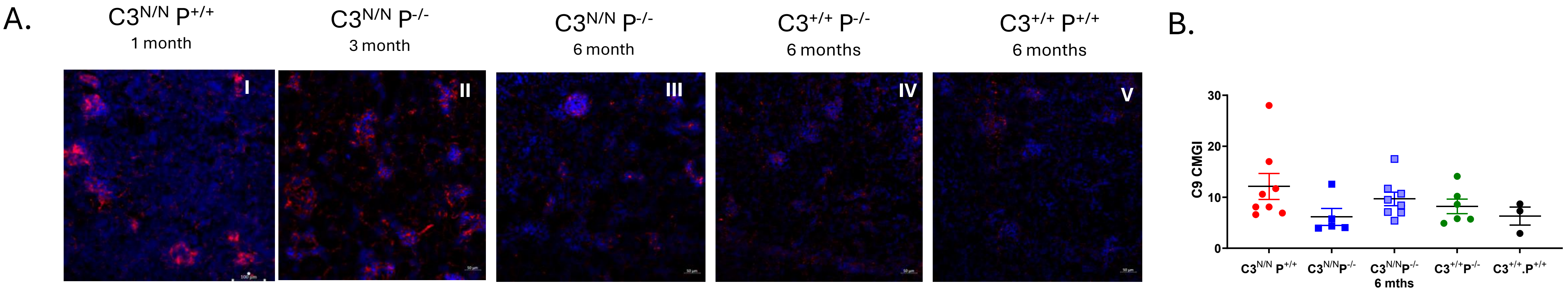

Glomerular C9 staining levels are not significantly altered by properdin deficiency. (A) Kidneys embedded in OCT were frozen on dry ice. After fixation, sections were, permeabilised with triton X and blocked with rabbit serum. Slides were incubated with a Rb anti-rat C9 (kind gift from Prof B.P. Morgan) and Goat anti-Rb-Alexa555 (150078, Abcam, UK). Slides underwent repetitive washing in PBS and then imaged after being mounted in DAPI mounting medium. Images were taken at x20 on Leica DM2000 LED using a Leica DFC 7000 T camera. Densitometry analysis of glomerular C9 deposition was performed using image J. Representative figures shown (A I-V). This is presented as corrected total glomerular fluorescence (CTGF) = Integrated Density – (area of manually selected glomerulus x mean fluorescence of six background readings) and between 40-60 glomeruli were scored for each mouse (B). No significant changes are noted between the groups (Two-way ANOVA).

Supplementary Figure 6

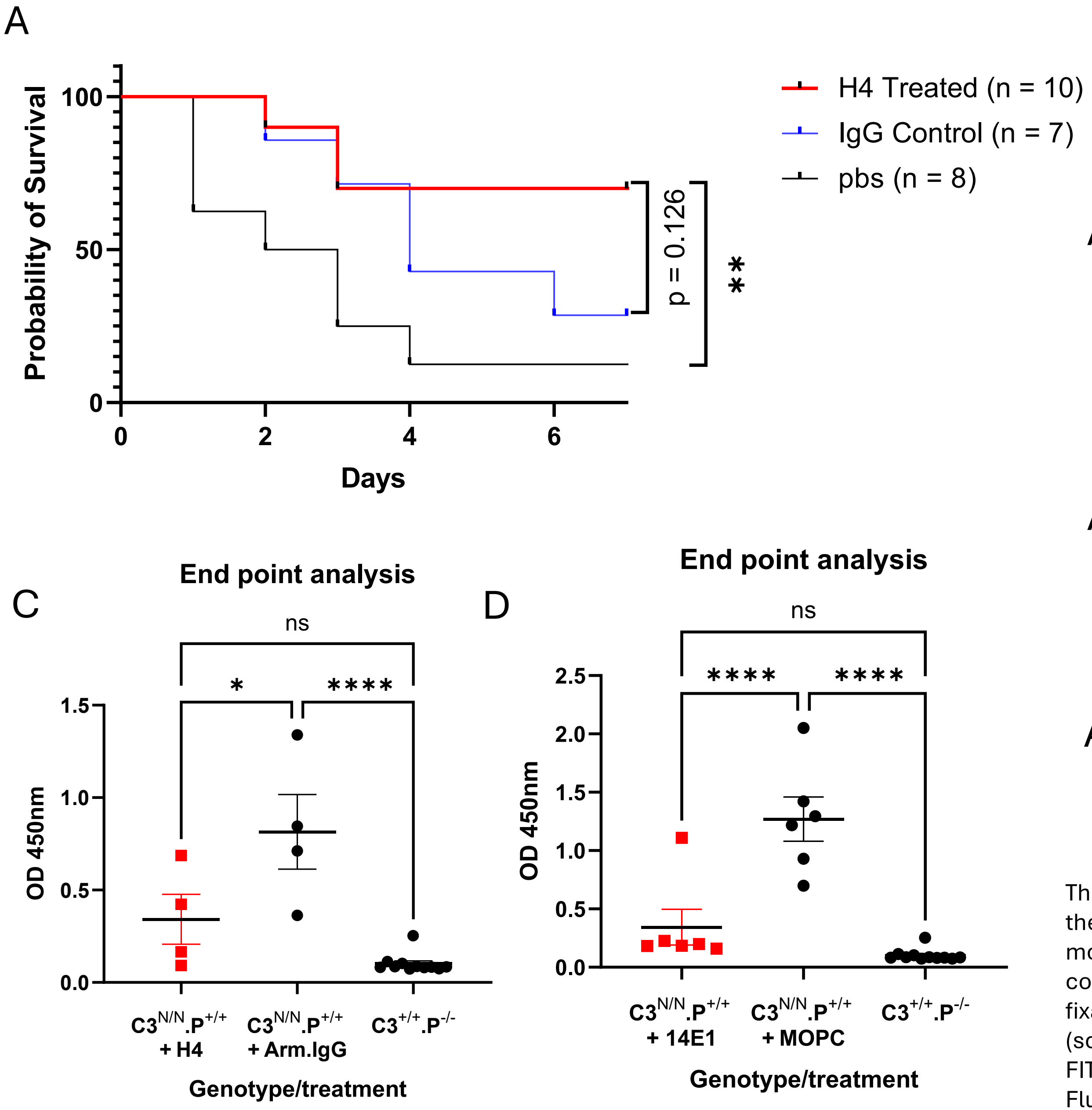

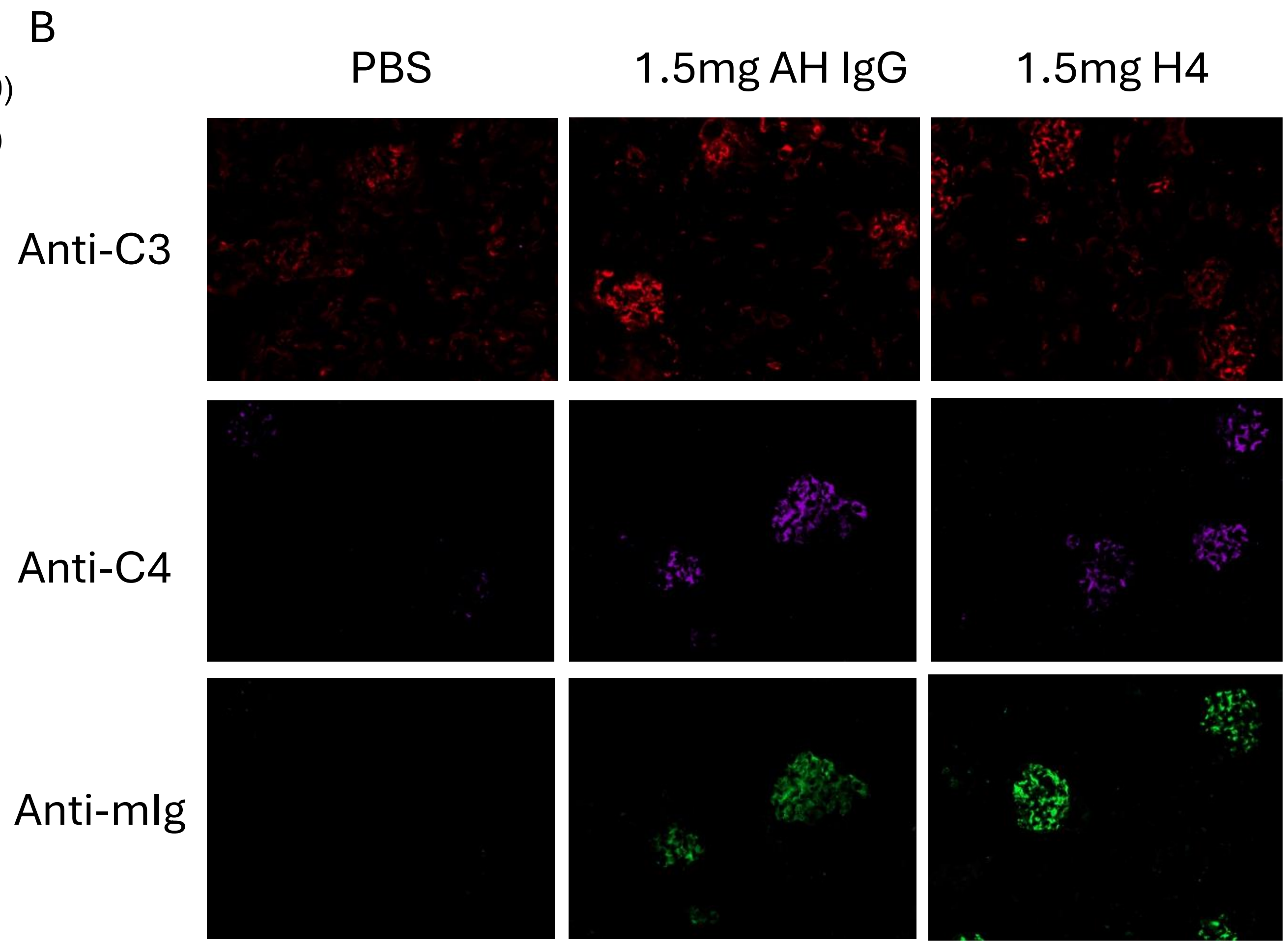

Therapeutic blockade of properdin using the hamster monoclonal antibody H4 is not significantly better than the Hamster IgG control, evidence of immune response to the hamster Ig limits utility of this approach in this model. (A) Absolute survival of H4 (n = 10), Hamster IgG (n = 7) or PBS treated (n = 8) C3N/N.P-/- following 2 consecutive days of haematuria (>25 ery/µl) (A). (B) Kidneys embedded in OCT were frozen on dry ice. After fixation, sections were blocked with goat serum. Slides were incubated with a goat anti-mouse C3 –Alexa647 (sc58926 AF647, clone 11H9, Santa Cruz, or rat anti-mouse C4 (Sigma, clone 16D2) or goat anti-mouse IgG-FITC (Sigma cat no F5387) diluted 1:200. As required, slides were then incubated with goat anti-rat IgG, Alexa Fluor™ 555 (Ab150158 Abcam, UK). Slides underwent repetitive washing in PBS and then imaged after being mounted in DAPI mounting medium. Images were taken at x20 on Leica DM2000 LED using a Leica DFC 7000 T camera. (C & D) Using the properdin ELISA described in supplemental figure 1a levels of free properdin were established in the cohorts of mice. One-way ANOVA. * $p<0.05$, **** $p<0.0001$, ns = not significant.
